# Effects of a High-Protein Diet Including Whole Eggs on Muscle Composition and Indices of Cardiometabolic Health and Systemic Inflammation in Older Adults with Overweight or Obesity: A Randomized Controlled Trial

**DOI:** 10.3390/nu10070946

**Published:** 2018-07-23

**Authors:** Christian S. Wright, Jing Zhou, R. Drew Sayer, Jung Eun Kim, Wayne W. Campbell

**Affiliations:** 1 Department of Nutrition Science, Purdue University, 700 W State Street, West Lafayette, IN 47907, USA; wrighch@iu.edu (C.S.W.); hattie517844@gmail.com (J.Z.); DREW.SAYER@ucdenver.edu (R.D.S.); chmkje@nus.edu.sg (J.E.K.); 2 Department of Physical Therapy, School of Health and Rehabilitation Sciences, Indiana University, Indianapolis, IN 46202, USA; 3 University of Colorado Anschutz Health and Wellness Center, University of Colorado Anschutz Medical Campus, 12348 E Montview Blvd, Aurora, CO 80045, USA; 4 Food Science & Technology Program, c/o Department of Chemistry, National University of Singapore, Science Drive 2, Singapore 117546, Singapore

**Keywords:** dietary protein, eggs, diet composition, skeletal muscle composition, intermuscular adipose tissue, inflammation

## Abstract

Age-related increases in intermuscular adipose tissue (IMAT) impair muscle quality, decrease functional capacity, and promote several cardiometabolic and inflammatory disorders. Whether these age-related alterations in muscle composition improve by consuming a high-protein (HP) diet with whole eggs are unclear. This parallel-design, randomized-controlled trial assessed the effects of a 12-week eucaloric HP diet with three whole eggs per day (1.4 g protein kg^−1^ day^−1^) versus a normal-protein diet void of eggs (NP, 0.8 g protein kg^−1^ day^−1^) on muscle composition (IMAT), cardiometabolic health, and systemic inflammation in older adults with overweight or obesity (12 men and 10 women; age 70 ± 5 years, BMI 31.3 ± 3.2 kg/m^2^, mean ± SD). No changes in muscle composition were observed over time, independent of protein intake. Total body weight was reduced in both groups (−3.3 ± 1.2%) and lean mass was preserved only with the HP diet. LDL concentration and hip circumference decreased only with the NP diet, while MCP-1 and HsCRP concentrations increased over time in both groups. A HP diet with whole eggs promotes lean mass retention with modest weight loss, but does not positively influence muscle composition, cardiometabolic health or systemic inflammation, compared to a NP diet void of eggs.

## 1. Introduction

Ectopic adipose tissue accumulation is an important hallmark of metabolic dysfunction and chronic disease. Obesity is strongly associated with metabolic and inflammatory disorders and recent evidence supports the anatomical location of adipose tissue [1,2,3,4,5] and its metabolic type [6] as determinants of the development of obesity-related disorders [7,8,9,10]. While gynoid adiposity (accumulation of adipose tissue in the thigh area) may be cardio-protective [10,11], adipose tissue located at ectopic sites like skeletal muscle, liver, or the intra-abdominal cavity are strongly associated with chronic inflammation [7,12], impaired glucose tolerance [1,2,3,13] and hyperlipidemia [5,14]. In particular, intermuscular adipose tissue (IMAT), adipose tissue located beneath the fascia and between muscle fibers and groups, impairs muscle quality and reduces insulin-stimulated skeletal muscle glucose uptake [15,16]. As such, increased IMAT is associated with insulin resistance [2,3,5,10,17], systemic inflammation [18,19], and skeletal muscle dysfunction, which is characterized by decreased muscle strength [20,21,22,23,24], function [25,26,27], and mobility [22,26,27,28].

Exercise and/or energy restriction are currently the two primary strategies for decreasing IMAT, with the greatest improvements in muscle composition occurring with a ≥10% decrease in body weight [29,30,31,32,33]. However, the effect of diet composition on IMAT concentrations in the absence of weight loss or with only modest weight loss has received little attention. This is of particular importance for older adults, a population prone to IMAT accumulation, where excessive weight loss may further impair muscle health and increase the symptoms of sarcopenia [34]. One potentially effective dietary strategy for decreasing IMAT in older adults is increasing total protein intake. Higher protein intakes are associated with improvements in body composition [35,36], glucose tolerance [37], inflammatory status [38,39], and postprandial lipemia [40,41].

Eggs are a nutrient dense whole-food source of dietary protein and antioxidants. Egg protein is highly bioavailable and able to effectively stimulate muscle protein synthesis [42]. Eggs are also a rich source of lutein and zeaxanthin [39]. Consuming 3 whole eggs per day for 12 weeks increased plasma concentrations of these carotenoids and adiponectin, which reduced systemic inflammation [39]. Adiponectin promotes the reduction of catabolic inflammatory cytokines and is inversely correlated with muscle fat storage [43,44,45]. A high-protein diet containing whole eggs may therefore reduce IMAT by decreasing overall adiposity, circulating postprandial fatty acids and inflammatory cytokines, and skeletal muscle lipid uptake [39,43].

The objective of this randomized controlled trial was to assess the effects of a high-protein diet with whole eggs, versus a normal protein diet void of eggs on muscle composition in older adults with overweight or obesity; with secondary endpoints including clinical indexes of cardiometabolic health and systemic inflammation. It was hypothesized that consumption of a high-protein diet with whole eggs for 12 weeks would improve muscle composition, cardiometabolic status, and systemic inflammation markers compared to consuming a normal protein diet void of eggs.

## 2. Materials and Methods

### 2.1. Participant Characteristics and Design

**Figure 1:**
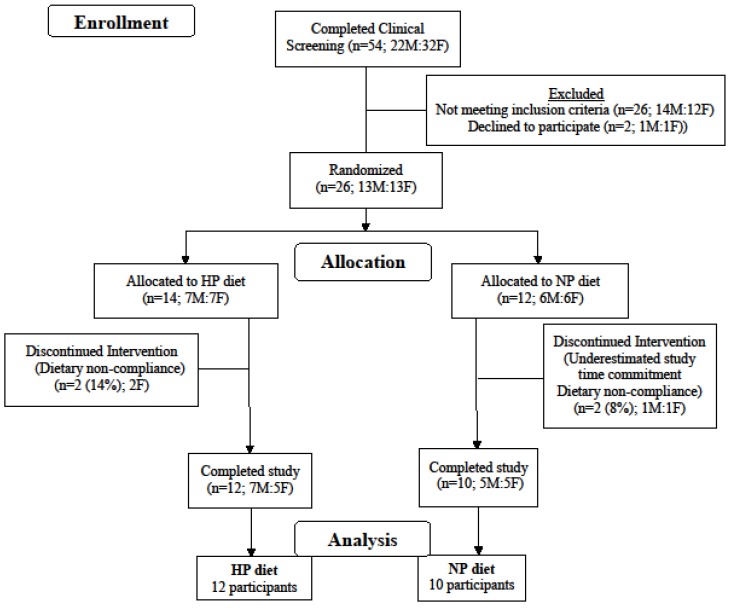
Consolidated Standards of Reporting Trials flow diagram. Flow of participants and study analysis. Females, F; High protein, HP; Males, M; Normal protein, NP.

Twenty-two adults were randomized to consume either a high-protein diet (HP) or a normal protein diet (NP) for 12 weeks (Figure 1). The inclusion criteria were as follows: male and female, age 50–80 years old; BMI 25–38 kg/m^2^; weight stable (±3 kg) during last 3 months; not currently or within the past 3 months following an exercise or weight loss program; non-diabetic; fasting glucose <110 mg/dL; blood pressure <160/100 mmHg; plasma total cholesterol <260 mg/dL; LDL-cholesterol <160 mg/dL; triacylglycerol <400 mg/dL; markers of kidney, liver, and heart functions within 10% of clinical normalcy. Participants habitually (≥3 months) consuming one or more medications for hypothyroidism (HP = 1, NP = 2), high blood pressure (HP = 6, NP = 3), and/or high cholesterol (HP = 4, NP = 2) were not excluded from the study. The study complied with the Declaration of Helsinki as revised in 2013 and the protocol received approval from the Biomedical Institutional Review Board at Purdue University. Each participant signed an informed consent document and received a monetary stipend for participation. The study is registered at clinicaltrials.gov as NCT01396915.

### 2.2. Dietary Intervention

Participants were randomly assigned (Microsoft Excel 2010, randomization function), to 1 of 2 dietary interventions: (1) a HP diet (*n* = 12) with 1.4 g protein kg^−1^ day^−1^; ~27% of energy from protein, 43% carbohydrate and 30% from fat, or (*2*) a NP diet (*n* = 10) with 0.8 g protein kg^−1^ day^−1^; ~15% of energy from protein, 55% carbohydrate and 30% from fat. The HP diet included ~50 g/day of additional dietary protein in comparison to the NP diet over the course of breakfast, lunch and mid-afternoon snack meals. The majority (59%) of the additional dietary protein came from whole eggs (3 eggs/day) and other egg products. Additional protein also came from higher dairy (26%), meat (20%), and plant (2%) sources, along with modestly reduced protein intakes from grains (−1%) and miscellaneous (−6%) sources. Participants consuming the HP diet were provided and asked to completely consume 3 whole eggs at breakfast, providing 20 g of dietary protein, and an egg-based afternoon snack, providing an additional ~10 g of dietary protein. Participants consuming the NP diet were restricted from eating eggs and egg products throughout the intervention but were provided and consumed isocaloric non-egg items at breakfast and as an afternoon snack. Each participant was provided and required to follow a rotating biweekly menu specific to their dietary group and daily energy requirement was estimated using sex-specific equations from the Institute of Medicine [46] for adults with overweight and obesity for the purpose of maintaining body weight. Within-day dietary protein intake was ≥20 g at four eating occasions for the HP diet compared to one eating occasion per day in the NP diet. An example of the daily protein intake and protein source distribution of each meal for a representative 85 kg participant averaged over a week for each diet is presented in Table 1.

Three 24-hour dietary recalls were collected on two weekdays (in-person) and one weekend day (phone interview) by either a registered dietitian or trained clinical research technician to assess the participants’ usual dietary intake before the start of the study. Participants completed daily menu checklists to document adherence to the diet during the 12-week intervention. Checklists were checked on a weekly basis by a registered dietitian or trained clinical research technician following food pick-up. Blood urea nitrogen (BUN) was assessed during the study as a crude marker of protein intake and dietary compliance at study weeks 0 (baseline), 4, 8, and 12. To promote body weight maintenance throughout the 12-week intervention, adjustments to the participant’s non-protein energy intakes were made if baseline body weight changed by >2 kg.

### 2.3. Muscle Composition

Medial muscle cross-sectional areas (MCSA), muscle volumes and IMATs of the thigh and calf were accessed by Magnetic Resonance Imaging (MRI). IMAT includes inter-muscular adipose tissue that resides between muscle groups and under the muscle fascia as well as intra-muscular adipose tissue that is located within muscles [47]. MRI’s of the thigh and calf muscles were obtained using a 3T General Electric (Waukesha, WI, USA) Signa HDx system at the Purdue MRI Facility. Following a 1-hour supine rest period to control for posture-related fluid shifts in muscle [48], participants were asked to lie with their heels in a fixed position on a nonmetallic support to control both the joint and scan angle while at the same time minimizing the compression of the legs. Following a localizer scan to ascertain the initial imaging position of the legs, a fast gradient echo sequence (TR/TE = 660/6.1 ms; FOV = 48 cm; acquisition matrix = 384 × 336; slice thickness = 6 mm; 60 axial slices) was performed. Muscle and adipose tissue volumes and MCSA were visualized and quantified using the analysis software MIPAV (Medical Image Processing, Analysis & Visualization, v. 7.0; Center for Information Technology, National Institutes of Health, Bethesda, MD, USA). The IMAT located between and within muscles was differentiated from subcutaneous adipose tissue by tracing the facial plane around the thigh by a trained member of the research staff. A Shading Correction algorithm (Inhomogeneity N3 correction) was applied followed by a Segmentation algorithm (Fuzzy Means, Single Channel) to quantify IMAT and muscle volume of each slice. Beginning with the appearance of the rectus femoris and ending with the appearance of the gluteus maximus, every third axial slice of the medial thigh was quantified in triplicate for MCSA, muscle volume and IMAT, which were then averaged and summed to calculate total thigh volumes. A similar protocol was carried out in the quantification of calf MCSA, muscle volume and IMAT, beginning with the appearance of the peroneus longus and ending with the disappearance of the gastrocnemius. Due to involuntary movement, which impaired tissue quantification, reduced sample sizes for medial thigh (HP (*n* = 9), NP (*n* = 8)) and medial calf (HP (*n* = 8), NP (*n* = 7)) are included in these analyses.

### 2.4. Whole Body Composition

Fasting-state body mass (total mass-robe mass) was measured every week using a digital platform scale (model ES200L, Ohaus Corporation, Pine Brook, NJ, USA) and standing height without shoes was measured at baseline using a wall-mounted stadiometer. Body mass index was calculated as (kg/m^2^) from these measurements. Waist circumference was measured in the standing position at the narrowest position between the lateral lower rib and the iliac crest. Hip circumference was measured in the standing position at the largest circumference of the lower abdomen. Waist and hip measurements were performed in triplicate at baseline and post-intervention and the time-specific mean values were recorded. Whole body and regional lean tissue and fat masses were also measured at baseline and post-intervention using dual-energy X-ray absorptiometry (DXA; GE Lunar Prodigy with version 11.1 enCORE iDXA software, Madison, WI, USA). Automatic daily calibrations of DXA imaging were conducted throughout the study and weekly quality-assurance tests were conducted utilizing a calibrated phantom spine.

### 2.5. Clinical Health Assessments

At baseline and post-intervention, blood samples were collected from an antecubital vein following an overnight fast and placed in tubes containing a clot activator to obtain serum or sodium heparin to obtain plasma (BD Vacutainer Brand; Becton, Dickinson and Co., Franklin Lakes, NJ, USA). Serum samples were sent to Mid-American Clinical Labs (Indianapolis, IN, USA) for analysis (comprehensive metabolic panel and lipid-lipoprotein panel). Plasma tubes were immediately placed on ice for 30 mins and centrifuged at 4 °C for 15 min at 4400 rpm. Aliquots of plasma were stored in microcentrifuge tubes at −80 °C for subsequent Cardiac High Sensitive C-reactive protein (HsCRP), tumor necrosis factor alpha (TNFα), interleukin 6 (IL-6), monocyte chemotactic protein 1 (MCP-1), insulin-like growth factor 1 (IGF-1) and adiponectin analyses. Blood pressure was measured in duplicate in the supine and reclining position following a 15-minute resting period using an electronic sphygmomanometer (Omron, Model # BP785; Kyoto, Japan) at baseline and the post-intervention.

### 2.6. Blood Analyses

Fasting plasma HsCRP was measured on a COBAS Integra 400 analyzer (Roche Diagnostic Systems) with a within-run precision coefficient of variance (CV) of 1.3% and a functional sensitivity (limit of quantitation) of 0.3 mg/L (2.96 nmol/L). Fasting plasma TNFα and IL-6 were determined by sandwich enzyme-linked immunosorbent assay (ELISA, Cayman Chemical Company, Ann Arbor, MI, USA) according to the manufacturer’s instructions with the following assay parameters: TNFα, intra-assay CV (5.7–9.2%), inter-assay CV (9.8–10.4%), detection limit (3.9 pg/mL); IL-6, intra-assay CV (4.1–5.1%), inter-assay CV (5.4–7.3%), detection limit (7.8 pg/mL). Fasting plasma MCP-1, IGF-1 and adiponectin were determined by sandwich ELISA (Sigma-Aldrich, St. Louis, MO, USA) according to the manufacturer’s instructions with an intra-assay and inter-assay CV of <10% and <12% and detection limits of <2 pg/mL, <2 ng/mL, and <25 pg/mL, respectively. All samples were measured in duplicate and absorbance was measured at 405 nm (TNFα, IL-6) or 450 nm (MCP-1, IGF-1, adiponectin) on an absorbance microplate reader (BioTeck EL × 808, Winooski, VT, USA).

### 2.7. Sample Size Estimation

At the time of study conception, we were unable to find a suitable study, one without an exercise or weight loss component, from which to derive statistical power calculations for our primary outcome of interest (IMAT). The Schrauwen-Hinderling et al. study revealed that intramyocellular lipid (IMCL) content increased following a 1-week high fat vs low fat diet (9.40 ± 1.87 vs. 6.12 ± 0.94 mmol/kg muscle wet weight, effect size = 2.2), demonstrating that diet can induce changes in skeletal muscle fat storage [49]. Due to considerable differences in study designs and indices of skeletal muscle composition (IMAT vs. IMCL), the current study was powered (80% power, α = 0.05) to detect a difference in IMAT concentration between diets that is equal to one standard deviation of the change (effect size = 1.0). Power calculations were also derived for changes in serum triglyceride concentrations as a secondary outcome of interest. Based upon a 16-week amino acid supplementation trial in older adults [50], 9 participants per group are needed to confirm a differential response of 0.30 mmol/L with >90% power (two-tailed, *p* < 0.05).

### 2.8. Statistical Analysis

Data distributions were reviewed and outliers were excluded according to the outlier labeling rule [51]. Baseline and post-intervention differences between groups were assessed using independent *t*-tests. Biological sex was established to be a significant covariant for multiple outcomes of interest. Effect of time, independent of diet and sex, on cardiometabolic outcomes was determined by paired *t*-test. Repeated-measures ANCOVA, controlling for sex, were performed to determine the main effects of diet, time, and diet-by-time interactions on muscle composition, cardiometabolic health, and systemic inflammation parameters. Significant diet-by-time interactions were followed up in post-hoc analyses through the comparison of simple main effects using Bonferroni correction. Significance was denoted with a corrected *p* value < 0.05. All statistical analyses were performed using SPSS statistical software (version 21; IBM Corporation 2012, Armonk, NY, USA). Results are presented as mean ± standard deviation.

## 3. Results

### 3.1. Participant Characteristics

Baseline participant characteristics are presented in Table 2. No differences in muscle and body compositions, anthropometrics, serum markers of fasting glucose, insulin, blood lipids, or macronutrient composition were observed between the HP and NP groups at baseline. Collectively, daily menu checklists indicated a compliance of 91% to the dietary intervention. BUN concentrations increased over time (*p* = 0.035) and were higher post-intervention with the HP diet versus the NP diet (*p* = 0.013). The BUN results provide a crude objective assessment of dietary protein intakes. Apparently, there were no differences in protein intakes of NP vs. HP at baseline, and no change in protein intake over time for NP. In contrast, protein intake increased over time for HP, and was higher for HP vs. NP at post-intervention.

### 3.2. Muscle Composition

MRI measurements of mid-thigh or mid-calf muscle composition were largely unaffected over the 12-week intervention or between dietary groups (Table 3). Subcutaneous fat to muscle volume ratio, however, decreased over time at the mid-calf with HP diet but not the NP diet (*p* = 0.031).

### 3.3. Whole Body Composition

**Figure 2:**
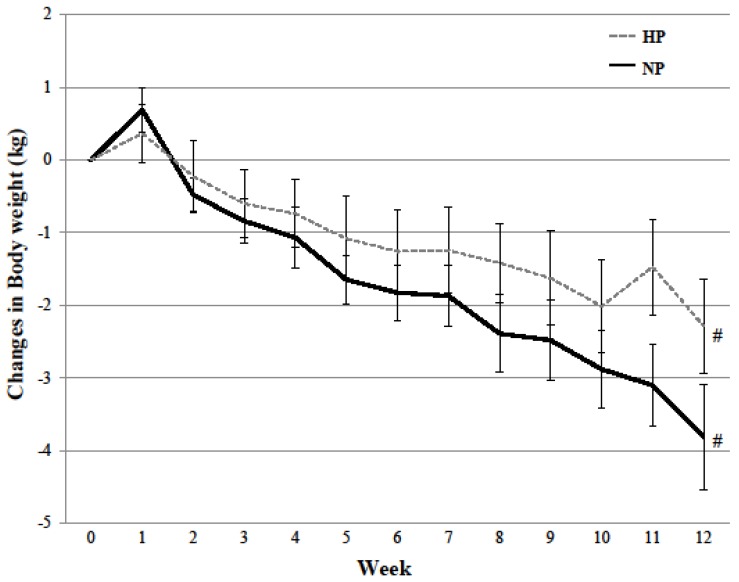
Changes in body weight over 12-week dietary intervention between the high protein and normal protein diet. Mean ± SD; Repeated measures ANCOVA controlling for sex: Time Effect (^#^ *p* values < 0.05). High protein, HP; Normal protein, NP.

Body weight (−3.00 ± 2.43 kg, *p* = 0.022, partial *r*^2^ = 0.246; Figure 2) and body fat (−2.25 ± 1.67 kg, *p* = 0.011, partial *r*^2^ = 0.266; Table 4) were reduced in both HP and NP diet groups over the 12-week dietary intervention. Changes in total lean mass (group × time, *p* = 0.05, partial *r*^2^ = 0.182), trunk lean mass (group × time, *p* = 0.015, partial *r*^2^ = 0.274), and appendicular fat mass (group × time, *p* = 0.033, partial *r*^2^ = 0.219) over time differed between the two diets. The HP diet prevented changes in lean mass (trunk lean mass (*p* = 0.568); total lean mass (*p* = 0.391)), while the NP diet decreased lean mass over the 12-week intervention (trunk lean mass (*p* = 0.006); total lean mass (*p* = 0.002); Table 4). Though both diets decreased appendicular fat mass over time, greater decreases were observed with the NP diet in comparison to the HP diet (*p* = 0.033, partial *r*^2^ = 0.219; Table 4).

### 3.4. Indices of Cardiometabolic Health

Independent of diet, IGF-1 concentrations increased over time *(p* = 0.042, partial *r*^2^ = 0.262) with a trend for an increase in the HP diet but no change in the NP diet (*p* = 0.082; Table 5). LDL concentration (*p* = 0.015, partial *r*^2^ = 0.274) and hip circumference (*p* = 0.003, partial *r*^2^ = 0.450) decreased over time with the NP diet but were not different from baseline with the HP diet (Table 5). Diet did not influence fasting glucose and insulin concentrations or other markers of cardiometabolic health (Table 5).

### 3.5. Systemic Inflammation

Diet did not influence plasma inflammatory markers (Table 6). Independent of diet, MCP-1 concentration (*p* = 0.028, partial *r*^2^ = 0.253) and HsCRP concentration (*p* = 0.011, partial *r*^2^ = 0.341) increased over time.

## 4. Discussion

Contrary to the hypothesis, a 12-week HP diet with whole eggs did not improve muscle composition, cardiometabolic health, or systemic inflammation in older adults with overweight and obesity. In the absence of ≥10% weight loss or exercise training, diet-induced changes in muscle composition (e.g., IMAT)—the primary outcome of interest—have rarely been addressed in the literature. Research showing that increased total protein intake improves whole body composition [35,36] and decreases elevated serum concentrations of numerous “IMAT contributors” including glucose [37], free fatty acid, chylomicron, triglyceride [40,41], and pro-inflammatory cytokines [38,39] provided scientific foundations for this study. Specifically, it was hypothesized that increased total protein intake would improve muscle composition in older adults, a population prone to IMAT accumulation [4] and muscle loss [52].

Changes in muscle composition were minimal over the 12-week intervention, showing no effect of dietary protein on muscle composition in older adults. Though a decrease in calf subcutaneous fat to muscle volume ratio was observed with the HP diet (*p* = 0.031), this alteration in fat to muscle volume ratio is likely a result of a decrease in subcutaneous fat (*p* = 0.160) versus a change in IMAT (*p* = 0.780) or muscle volume (*p* = 0.795) by the HP diet. There is a paucity of data on the effects of dietary protein, within the context of a diet-only intervention (e.g. weight maintenance or diet-induced modest weight loss), on muscle composition in older adults with overweight or obesity. Protein supplementation (10 g/day) was associated with increased thigh muscle cross-sectional area (MSCA) in bedridden, frail older adults (>70 years), whereas the current study population represents the average US older adult who is still active, independent, and not severely protein-malnourished [53]. Therefore, current results suggest that higher dietary protein intake for 12 weeks does not influence muscle composition in healthy older adults with overweight or obesity.

The 12-week dietary intervention did, however, alter the participants’ normal dietary habits and change body composition. Independent of diet, biological sex, and despite increases in the participant’s non-protein energy intake to maintain body weight, decreases in waist circumference, total body mass, total fat mass, and total lean mass were observed over time. Such modest weight loss often occurs following prescribed, fixed meal diets [54,55]. While in general, weight loss is associated with improvements in health [56], the current study’s 3% decrease in body mass (−3.00 ± 2.43 kg) did not affect the majority of study outcomes. Protein intake did, however, augment changes in body composition following modest weight loss, where the HP diet prevented the loss of lean mass observed with the NP diet over the 12-week intervention. This attenuation of lean mass following modest weight loss is consistent with the literature and showcases dietary protein’s ability to preferentially decrease fat mass over lean mass. A recent systematic review and meta-analysis of 24 randomized controlled trials supports this claim. In accordance with PRISMA guidelines and following the accumulation over 1500 articles from 4 different databases, meta-analyses revealed that higher protein diets (>1.0 g protein kg^−1^ d^−1^) attenuated more lean mass (+0.83 kg) and reduced more fat mass (−0.53 kg) in older adults (>50 years, *N* = 242) in comparison to normal protein diets (<1.0 g protein kg^−1^ d^−1^) [57]. The current study further supports this effect of dietary protein to retain lean mass during modest weight loss in older adults. The preservation of lean mass is essential for health aging, as age-related decreases in lean mass are associated with impairments in mobility [25,58], decreased independence [59], and an increased risk of all-cause mortality [60]. Collectively, a higher protein diet can help maintain lean mass in older adults with overweight or obesity following modest weight loss and increasing whole egg consumption provides a viable whole foods approach to increase total protein intake.

Contrary to the hypothesis, only the NP diet influenced cardiometabolic health, decreasing LDL concentration over the 12-week intervention. Consuming an egg-free NP diet for 12 weeks decreased LDL concentration (−0.3 ± 0.3 mmol/L) in older adults with overweight and obesity, while the HP diet, where at least 3 whole eggs were consumed per day, did not influence any marker of cardiometabolic health. Though contrary to our hypothesis, these results are consistent with recent findings [61,62,63] and show that consuming whole eggs does not influence total cholesterol, LDL concentrations [62], endothelial function, blood pressure [61], nor atherosclerosis [63] in at risk adults with overweight and obesity. Furthermore, whether the observed decrease in LDL concentrations with the NP diet equates to improvements in cardiovascular health, particularly in individuals at intermediate cardiovascular risk like the current study participants [64], has come under question in recent years [65]. Despite statins proving effective in lowering LDL concentrations, many patients continue to have clinical events following its reduction [66]. Therefore, recent recommendations suggest lipoprotein ratios or “atherogenic indices” have a greater predictive capacity for cardiovascular events in comparison to a single lipoprotein [67]. Thus, caution is given in the interpretation of the study’s lipoprotein results as the total cholesterol to HDL ratio did not change in both diets over the 12-weeks.

The current study extends literary knowledge by investigating the influence of diet composition on changes in IMAT; assessing whether a diet-induced, whole-foods approach could decrease IMAT without extensive weight loss or exercise training. Strengths of this study include the use of MRI modality to assess medial thigh and calf muscle composition, DXA to assess changes in whole-body composition, and the measurement of both cardiometabolic and inflammatory outcomes. There were some limitations to the current study. Involuntary participant movement during the baseline and post-intervention MRI data acquisition reduced thigh (*n* = 17) and calf (*n* = 15) MRI sample sizes allotted for muscle composition analysis. Involuntary participant movement or muscle spasms during MRI data acquisition is often the result of peripheral neuromuscular simulation as oscillating magnetic field gradients can stimulate the peripheral nervous system and cause involuntary muscle spasms in healthy adults [68,69]. Therefore, if involuntary movement persists following additional attempts, researchers should determine the appropriate imaging correction techniques to remove motion artifacts [70,71]. Some may also consider the study’s modest weight loss (−3.3%) as a limitation, potentially confounding results. However, considering weight-loss induced decreases in the primary outcome interest (IMAT) seems to require at least a 10% decrease in body mass [29,30,31,32,33] and the vast majority of secondary outcomes were unaffected by a similar amount of weight loss in both groups, the study’s modest weight loss is unlikely to have influenced final interpretation. Post hoc power calculations indicated that the variability in changes in IMAT following the HP and NP diets were greater than anticipated, which may have limited the ability to detect diet-induced changes in IMAT with the current sample size. However, the current study is among the first to study diet-induced changes in IMAT, and currently the only study to investigate the effects of dietary protein on IMAT without exercise training or ≥10% weight loss. Thus, the current study provides valuable results for future investigators when making statistical power calculations.

## 5. Conclusions

For older adults with overweight and obesity, consumption of a high-protein diet with whole eggs for 12 weeks promotes the retention of lean mass following modest weight loss. However, increasing total protein intake and consuming three whole eggs per day does not provide an effective dietary strategy to positively influence skeletal muscle composition, including IMAT, or indices of cardiometabolic health and systemic inflammation, in comparison to a normal protein diet void of eggs.

## Figures and Tables

**Table 1:** Average daily protein intake and source distribution for a representative 85 kg adult participant ^1^.

	Total	Egg	Meat	Plant	Grain	Dairy	Residual
NP							
Breakfast, g	12.7	0.0	3.0	0.6	7.0	1.1	0.9
Lunch, g	12.6	0.0	2.7	0.6	4.5	2.8	1.9
Dinner, g	31.7	0.0	15.0	1.6	6.5	6.2	2.4
Snack, g	12.5	0.0	0.0	3.5	0.0	7.7	1.3
Total Intake, g	69.5	0.0	20.7	6.3	18.1	17.8	6.5
Percentage, %	100%	0%	30%	9%	26%	26%	9%
HP							
Breakfast, g	30.0	12.5	6.2	1.3	7.6	1.8	0.6
Lunch, g	31.2	6.3	8.1	2.3	5.6	8.6	0.3
Dinner, g	32.4	0.0	16.2	1.5	4.6	7.9	2.2
Snack, g	25.8	10.6	0.0	2.2	0.0	12.5	0.5
Total Intake, g	119.4	29.4	30.5	7.4	17.8	30.8	3.5
Percentage, %	100%	25%	26%	6%	15%	26%	3%
Added Protein (HP-NP)							
Breakfast, g	17.3	12.5	3.2	0.7	0.6	0.7	−0.3
Lunch, g	18.6	6.3	5.4	1.7	1.1	5.8	−1.6
Dinner, g	0.7	0.0	1.2	−0.1	−1.9	1.7	−0.2
Snack, g	13.3	10.6	0.0	−1.3	0.0	4.8	−0.8
Total Intake, g	49.9	29.4	9.8	1.1	−0.3	13.0	−3.0
Percentage, %	100%	59%	20%	2%	−1%	26%	−6%

^1^ HP, High protein; NP, Normal protein.

**Table 2:** Baseline participant characteristics ^1^.

Variable	All	HP	NP
*n*	22	12	10
Age, years	70 ± 5	70 ± 6	71 ± 3
Male:Female	12:10	7:5	5:5
Body mass, kg	92.1 ± 15.4	90.1 ± 16.4	91.0 ± 16.1
Height, cm	171 ± 11	167 ± 12	172 ± 9
BMI, kg/m ^2^	31.3 ± 3.2	32.2 ± 3.4	30.5 ± 3.3
Waist, cm	110 ± 9	110 ± 11	110 ± 9
Hip, cm	115 ± 9	114 ± 11	115 ± 9
Lean mass, kg	51.3 ± 10.9	49.0 ± 11.6	50.7 ± 10.3
Fat mass, kg	37.2 ± 8.4	37.6 ± 9.2	36.9 ± 8.9
Percentage body fat	42.1 ± 7.3	43.5 ± 8.3	42.0 ± 6.7
Fasting glucose, mg/dL	99.2 ± 9.7	98.1 ± 11.1	98.3 ± 10.0
Fasting insulin, µU/mL	13.1 ± 7.5	11.4 ± 7.1	13.1 ± 7.2
HOMA-IR	3.3 ± 2.1	2.8 ± 2.1	3.3 ± 2.0
Total cholesterol, mg/dL	190.4 ± 37.0	201.4 ± 33.5	185.5 ± 26.7
LDL cholesterol, mg/dL	115.7 ± 32.4	123.5 ± 38.0	111.5 ± 18.2
HDL cholesterol, mg/dL	48.7 ± 14.6	54.0 ± 11.2	47.6 ± 18.1
Total Cholesterol:HDL	4.2 ± 1.2	3.9 ± 1.1	4.3 ± 1.5
Triglycerides, mg/dL	130.1 ± 58.6	119.9 ± 64.0	131.6 ± 64.7
Manual SBP ^3^, mm Hg	132 ± 14	129 ± 12	134 ± 15
Manual DBP ^3^, mm Hg	84 ± 7	81 ± 7	86 ± 8
BUN, mg/dL	16.5 ± 3.3	17.0 ± 4.6	16.2 ± 2.6
Total Energy, kcal/day	2011 ± 537	2061 ± 597	1957 ± 597
Total Fat, g/day	79 ± 31	82 ± 35	75 ± 35
Total Fat, %	34 ± 7	34 ± 6	33 ± 6
Total Carbohydrates, g/day	245 ± 69	249 ± 71	241 ± 71
Total Carbohydrates, %	48 ± 8	48 ± 6	48 ± 6
Total Protein, g/day	82 ± 24	84 ± 15	79 ± 15
Total Protein, %	17 ± 5	16 ± 4	17 ± 4
Animal Protein, g/day	54 ± 25	55 ± 12	53 ± 12
Vegetable Protein, g/day	28 ± 8	29 ± 6	27 ± 6

^1^ Mean ± SD; ^2^ BUN, Blood urea nitrogen; DBP, Diastolic blood pressure; HDL, High-density lipoprotein; HP, High protein; HOMA-IR, Homeostatic model assessment of insulin resistance; LDL, Low-density lipoprotein; NP, Normal protein; SBP, Systolic blood pressure. ^3^ Medication usage: hypothyroidism (HP = 1, NP = 2), high blood pressure (HP = 6, NP = 3), and/or high cholesterol (HP = 4, NP = 2).

**Table 3:** MRI-derived measurements of muscle composition ^1^.

		Baseline	Post	∆
Thigh		(*n* = 18)	(*n* = 19)	(*n* = 18)
MCSA, mm^2^ × 10^4^	NP	258.4 ± 54.0	218.9 ± 47.0	−29.2 ± 65.1
	HP	260.2 ± 69.4	254.4 ± 98.50	9.3 ± 114.0
	All	259.3 ± 60.3	237.6 ± 78.5	−8.8 ± 93.5
SubQ, mm^2^ × 10^4^	NP	104.8 ± 49.0	68.3 ± 36.4	−28.6 ± 33.8
	HP	82.1 ± 42.0	82.4 ± 35.9	1.8 ± 53.8
	All	93.5 ± 45.7	75.7 ± 45.7	−12.5 ± 46.8
IMAT, mm^3^ × 10^4^	NP	16.7 ± 11.3	12.5 ± 4.4	−4.9 ± 10.2
	HP	17.9 ± 7.8	15.1 ± 7.4	−2.0 ± 6.1
	All	17.3 ± 9.4	13.9 ± 6.1	−3.3 ± 8.2
Muscle Volume, mm^3^ × 10^4^	NP	119.5 ± 38.7	110.5 ± 45.3	−7.4 ± 28.3
	HP	151.4 ± 68.2	125.5 ± 61.1	−16.2 ± 60.5
	All	135.4 ± 56.3	118.4 ± 53.2	−12.1 ± 46.9
Total Fat:CSA	NP	0.47 ± 0.16	0.38 ± 0.16	−0.07 ± 0.06
	HP	0.40 ± 0.18	0.40 ± 0.15	−0.02 ± 0.10
	All	0.43 ± 0.17	0.39 ± 0.15	−0.04 ± 0.09
IMAT:Muscle Volume	NP	0.12 ± 0.04	0.12 ± 0.03	−0.03 ± 0.06
	HP	0.11 ± 0.03	0.13 ± 0.05	−0.01 ± 0.01
	All	0.12 ± 0.03	0.13 ± 0.04	−0.02 ± 0.04
SubQ:Muscle Volume	NP	1.03 ± 0.64	0.82 ± 0.72	−0.10 ± 0.28
	HP	0.78 ± 0.73	0.87 ± 0.64	−0.01 ± 0.31
	All	0.90 ± 0.68	0.85 ± 0.66	−0.05 ± 0.29
Calf		(*n* = 15)	(*n* = 17)	(*n* = 15)
MCSA, mm^2^ × 10^4^	NP	134.9 ± 28.8	143.6 ± 41.6	2.0 ± 15.5
	HP	142.0 ± 30.7	141.0 ± 27.2	−1.9 ± 4.8
	All	138.6 ± 29.0	142.2 ± 33.6	0.2 ± 11.6
SubQ, mm^2^ × 10^4^	NP	48.3 ± 41.6	49.8 ± 25.2	0.6 ± 2.7
	HP	48.8 ± 33.7	49.8 ± 31.9	−0.9 ± 2.0
	All	48.6 ± 30.3	49.8 ± 28.0	−0.1 ± 2.4
IMAT, mm^3^ × 10^4^	NP	9.3 ± 3.3	9.9 ± 4.6	−0.6 ± 1.3
	HP	10.0 ± 3.6	10.0 ± 3.6	−1.1 ± 1.0
	All	9.7 ± 3.4	9.9 ± 3.3	−0.9 ± 1.1
Muscle Volume, mm^3^ × 10^4^	NP	77.6 ± 21.7	80.5 ± 22.3	1.2 ± 6.9
	HP	79.6 ± 18.3	79.2 ± 18.3	−0.5 ± 3.0
	All	78.6 ± 19.3	79.8 ± 20.1	−0.2 ± 5.3
Total Fat: CSA	NP	0.44 ± 0.23	0.44 ± 0.22	0.01 ± 0.06
	HP	0.42 ± 0.25	0.43 ± 0.24	−0.01 ± 0.01
	All	0.43 ± 0.23	0.43 ± 0.22	−0.004 ± 0.05
IMAT:Muscle Volume	NP	0.12 ± 0.04	0.12 ± 0.05	−0.01 ± 0.02
	HP	0.12 ± 0.03	0.13 ± 0.03	−0.01 ± 0.01
	All	0.12 ± 0.03	0.13 ± 0.04	−0.01 ± 0.01
SubQ:Muscle Volume	NP	0.67 ± 0.39	0.66± 0.36	0.01 ± 0.02 ^A^
	HP	0.64 ± 0.43	0.68 ± 0.44	−0.01 ± 0.02 ^A,B^
	All	0.66 ± 0.40	0.67 ± 0.40	−0.002 ± 0.02

^1^ Mean ± SD; Repeated measures ANOVA controlling for sex: Diet-by-Time Interaction (^A^ *p* values < 0.05), Time Effect (^B^ *p* values < 0.05). HP, High protein; NP, Normal protein; MAT, Intramuscular adipose tissue; MCSA, Muscle cross-sectional area; MRI, Magnetic resonance imaging; SubQ, Subcutaneous fat.

**Table 4:** Body composition ^1^.

			Baseline	Post	∆
			(*n* = 22)	(*n* = 22)	(*n* = 22)
Appendicular fat mass, kg		NP	15.72 ± 4.23	14.68 ± 4.05	−1.04 ± 0.76 ^A^
		HP	14.84 ± 4.99	14.41 ± 4.94	−0.43 ± 0.52 ^A^
		All	15.24 ± 4.58	14.53 ± 4.45	−0.71 ± 0.70
Appendicular lean mass, kg		NP	23.34 ± 5.49	22.76 ± 5.56	−0.58 ± 0.66
		HP	23.61 ± 5.93	23.22 ± 5.90	−0.39 ± 0.71
		All	23.49 ± 5.60	23.01 ± 5.62	−0.48 ± 0.68
Trunk fat mass, kg		NP	20.13 ± 6.27	18.51 ± 6.32	−1.62 ± 1.21
		HP	21.64 ± 4.89	20.18 ± 5.05	−1.46 ± 1.16
		All	20.95 ± 5.47	19.42 ± 5.59	−1.53 ± 1.16
Trunk lean mass, kg		NP	23.98 ± 4.67	23.30 ± 4.58	−0.68 ± 0.72 ^A^
		HP	24.69 ± 5.49	24.81 ± 5.56	0.12 ± 0.63 ^A^
		All	24.37 ± 5.03	24.13 ± 5.08	−0.24 ± 0.77
Percent body fat		NP	41.99 ± 6.65	40.75 ± 6.77	−1.24 ± 1.26
		HP	42.20 ± 8.14	41.00 ± 8.26	−1.20 ± 1.04
		All	42.11 ± 7.33	40.88 ± 7.44	−1.22 ± 1.11
Total fat mass, kg		NP	36.83 ± 8.85	34.15 ± 8.98	−2.68 ± 1.81
		HP	37.50 ± 8.32	35.60 ± 8.65	−1.90 ± 1.52
		All	37.20 ± 8.37	34.94 ± 8.62	−2.25 ± 1.67 ^B^
Total lean mass, kg		NP	50.71 ± 10.33	49.42 ± 10.37	−1.29 ± 0.97 ^A,B^
		HP	51.80 ± 11.77	51.52 ± 11.76	−0.28 ± 1.20 ^A^
		All	51.30 ± 10.89	50.56 ± 10.94	−0.74 ± 1.19
Total mass, kg		NP	90.5 ± 15.8	86.49 ± 16.23	−3.96 ± 2.12
		HP	92.1 ± 15.4	89.89 ± 16.07	−2.20 ± 2.46
		All	91.3 ± 15.2	88.3 ± 15.8	−3.00 ± 2.43 ^B^

^1^ Mean ± SD; Repeated measures ANCOVA controlling for sex: Diet-by-Time Interaction (^A^ *p* values < 0.05), Time Effect (^B^ *p* values < 0.05). HP, High protein; NP, Normal protein.

**Table 5:** Indices of cardiometabolic health ^1^.

			Baseline	Post	∆
			(*n* = 22)	(*n* = 22)	(*n* = 22)
Waist, cm		NP	110 ± 9	102 ± 13	−8 ± 9
		HP	110 ± 11	107 ± 13	−3 ± 8
		All	110 ± 9	105 ± 13	−5 ± 9 ^B^
			*(n = 22)*	*(n = 22)*	*(n = 22)*
Hip, cm		NP	115 ± 9	111 ± 12	−5 ± 3 ^A,B^
		HP	114 ± 11	116 ± 9	1 ± 4 ^A^
		All	115 ± 9	114 ± 10	−2 ± 5
			*(n = 22)*	*(n = 22)*	*(n = 22)*
Fasting glucose, mmol/L		NP	5.5 ± 0.6	5.5 ± 0.4	0.0 ± 0.6
		HP	5.6 ± 0.5	5.4 ± 0.5	−0.1 ± 0.4
		All	5.5 ± 0.5	5.5 ± 0.5	−0.1 ± 0.5
			(*n* = 19)	(*n* = 22)	(*n* = 19)
Fasting insulin, mmol/L		NP	91.0 ± 50.0	82.6 ± 36.1	−8.3 ± 38.9
		HP	90.3 ± 57.6	62.5 ± 37.5	−35.4 ± 34.7
		All	91 ± 52.1	69.5 ± 36.8	−21.5 ± 38.2
HOMA-IR		NP	3.26 ± 1.98	2.92 ± 1.33	−0.35 ± 1.59
		HP	3.32 ± 2.43	2.23 ± 1.43	−1.37 ± 1.51
		All	3.29 ± 2.15	2.55 ± 1.40	−0.83 ± 1.60
			(*n* = 22)	(*n* = 22)	(*n* = 22)
Total cholesterol, mmol/L		NP	4.8 ± 0.7	4.6 ± 0.8	−0.2 ± 0.4
		HP	5.0 ± 1.2	5.0 ± 1.1	<−0.1 ± 0.6
		All	4.9 ± 1.0	4.8 ± 1.0	−0.1 ± 0.5
LDL cholesterol, mmol/L		NP	2.9 ± 0.5	2.6 ± 0.5	−0.3 ± 0.3 ^A,B^
		HP	3.1 ± 1.1	3.2 ± 1.0	0.1 ± 0.4 ^A^
		All	3.0 ± 0.8	3.0 ± 0.9	<−0.1 ± 0.4
HDL cholesterol, mmol/L		NP	1.2 ± 0.5	1.2 ± 0.5	<−0.1 ± 0.2
		HP	1.3 ± 0.3	1.2 ± 0.3	−0.1 ± 0.2
		All	1.3 ± 0.4	1.2 ± 0.4	−0.1 ± 0.2
Cholesterol: HDL		NP	4.33 ± 1.50	4.12 ± 1.27	−0.21 ± 0.56
		HP	4.04 ± 1.02	4.18 ± 0.95	0.14 ± 0.36
		All	4.17 ± 1.24	4.15 ± 1.08	−0.02 ± 0.48
Triglycerides, mmol/L		NP	1.5 ± 0.7	1.6 ± 0.8	0.1 ± 0.4
		HP	1.5 ± 0.6	1.2 ± 0.4	−0.3 ± 0.6
		All	1.5 ± 0.7	1.4 ± 0.6	−0.1 ± 0.5
Manual SBP ^3^, mm HG		NP	134 ± 15	132 ± 11	−2 ± 12
		HP	130 ± 14	123 ± 17	−7 ± 13
		All	132 ± 14	127 ± 14	−4 ± 13
Manual DBP ^3^, mm HG		NP	86 ± 8	84 ± 7	−2 ± 6
		HP	83 ± 6	78 ± 10	−5 ± 5
		All	84 ± 7	81 ± 9	−4 ± 6
BUN ^2^, mmol/L		NP	5.8 ± 0.9	5.6 ± 1.2 ^C^	−0.2 ± 1.2 ^A^
		HP	6.0 ± 1.4	7.6 ± 2.6 ^C^	1.6 ± 1.8 ^A,B^
		All	5.9 ± 1.2	6.7 ± 2.2	0.8 ± 1.8
			(*n* = 17)	(*n* = 17)	(*n* = 17)
IGF-1, pg/ml		NP	1.52 ± 0.99	1.37 ± 0.69	−0.15 ± 0.43
		HP	1.95 ± 1.71	2.82 ± 2.47	0.99 ± 1.81
		All	1.80 ± 1.47	2.31 ± 2.11	0.56 ± 1.53 ^B^

^1^ Mean ± SD; Repeated measures ANOVA controlling for sex: Diet-by-Time Interaction (^A^ *p* values < 0.05), Time Effect (^B^ *p* values < 0.05); Post Independent *t*-Test (^C^ *p* values < 0.05). ^2^ BUN, Blood urea nitrogen; DBP, Diastolic blood pressure; HDL, High-density lipoprotein; HP, High protein; HOMA-IR, Homeostatic model assessment of insulin resistance; IGF-1, Insulin-like growth factor 1; LDL, Low-density lipoprotein; NP, Normal protein; SBP, Systolic blood pressure. ^3^ Measured in the seated position.

**Table 6:** Inflammatory status ^1^.

		Baseline	Post	∆
		(*n* = 21)	(*n* = 20)	(*n* = 20)
HsCRP, mg/L	NP	2.25 ± 1.50	2.10 ± 1.77	−0.14 ± 0.97
	HP	1.32 ± 1.08	1.62 ± 1.49	0.30 ± 0.77
	All	1.72 ± 1.35	1.83 ± 1.64	0.11 ± 0.89 ^B^
		(*n* = 21)	(*n* = 20)	(*n* = 20)
TNFα, pg/mL	NP	2.93 ± 1.47	3.37 ± 1.31	1.06 ± 1.49
	HP	2.97 ± 1.06	4.52 ± 2.11	0.65 ± 1.99
	All	2.95 ± 1.24	4.00 ± 1.84	0.84 ± 1.74
		(*n* = 19)	(*n* = 19)	(*n* = 19)
IL-6, pg/mL	NP	3.55 ± 2.01	4.04 ± 2.50	1.04 ± 2.82
	HP	2.62 ± 1.04	3.36 ± 1.68	0.75 ± 1.56
	All	3.06 ± 1.60	3.38 ± 2.07	0.89 ± 2.20
		(*n* = 20)	(*n* = 20)	(*n* = 20)
MCP-1, pg/mL	NP	154.99 ± 42.42	175.45 ± 28.92	20.11 ± 45.26
	HP	164.22 ± 36.47	167.79 ± 49.30	3.57 ± 27.40
	All	160.07 ± 38.47	171.24 ± 40.58	11.44 ± 36.99 ^B^
		(*n* = 19)	(*n* = 20)	(*n* = 19)
Adiponectin, μg/mL	NP	9.48 ± 2.39	8.07 ± 3.44	−0.92 ± 3.20
	HP	11.76 ± 4.31	11.37 ± 5.63	−0.76 ± 4.62
	All	10.69 ± 9.72	9.72 ± 4.85	−0.83 ± 3.94

^1^ Mean ± SD; Repeated measures ANCOVA controlling for sex: Time Effect (^B^ *p* values < 0.05). HP, High protein; HsCRP, High-sensitivity C-reactive protein; IL-6, Interleukin 6; MCP-1, Monocyte chemoattractant protein 1; NP, Normal protein; TNFα, Tumor necrosis factor alpha.
